# Myostatin and activin blockade by engineered follistatin results in hypertrophy and improves dystrophic pathology in *mdx* mouse more than myostatin blockade alone

**DOI:** 10.1186/s13395-018-0180-z

**Published:** 2018-10-27

**Authors:** Andrea Iskenderian, Nan Liu, Qingwei Deng, Yan Huang, Chuan Shen, Kathleen Palmieri, Robert Crooker, Dianna Lundberg, Niksa Kastrapeli, Brian Pescatore, Alla Romashko, John Dumas, Robert Comeau, Angela Norton, Jing Pan, Haojing Rong, Katayoun Derakhchan, David E. Ehmann

**Affiliations:** 1 Research, Shire Pharmaceuticals, Lexington, MA 02421 USA; 2 Discovery Therapeutics, Shire Pharmaceuticals, Lexington, MA USA; 3 Nonclinical Development, Shire Pharmaceuticals, Lexington, MA USA; 4 Drug Discovery, Shire, Cambridge, MA USA

**Keywords:** Myostatin, Activin a, Follistatin, Hypertrophy, Fibrosis, *mdx*, Duchenne muscular dystrophy

## Abstract

**Background:**

Myostatin antagonists are being developed as therapies for Duchenne muscular dystrophy due to their strong hypertrophic effects on skeletal muscle. Engineered follistatin has the potential to combine the hypertrophy of myostatin antagonism with the anti-inflammatory and anti-fibrotic effects of activin A antagonism.

**Methods:**

Engineered follistatin was administered to C57BL/6 mice for 4 weeks, and muscle mass and myofiber size was measured. In the *mdx* model, engineered follistatin was dosed for 12 weeks in two studies comparing to an Fc fusion of the activin IIB receptor or an anti-myostatin antibody. Functional measurements of grip strength and tetanic force were combined with tissue analysis for markers of necrosis, inflammation, and fibrosis to evaluate improvement in dystrophic pathology.

**Results:**

In wild-type and *mdx* mice, dose-dependent increases in muscle mass and quadriceps myofiber size were observed for engineered follistatin. In *mdx*, increases in grip strength and tetanic force were combined with improvements in muscle markers for necrosis, inflammation, and fibrosis. Improvements in dystrophic pathology were greater for engineered follistatin than the anti-myostatin antibody.

**Conclusions:**

Engineered follistatin generated hypertrophy and anti-fibrotic effects in the *mdx* model.

## Background

Duchenne muscular dystrophy (DMD) is a monogenic, X-linked, progressive neuromuscular disease caused by mutations in the gene encoding dystrophin, a critical structural protein of skeletal, cardiac, and smooth muscle [1]. Dystrophin malfunction and consequent instability of the juncture between the extracellular matrix and internal cytoskeleton of muscle cells results in chronic deterioration of muscle strength and function. Patients with DMD typically lose ambulation in adolescence and succumb to cardiopulmonary failure in early adulthood [2]. The current pharmaceutical standard of care is corticosteroid treatment, although therapies that modulate dystrophin expression, such as exon-skipping oligonucleotides, have been approved on their promise to slow disease progression.

There is still a high remaining medical need in the armamentarium against DMD for drugs effective against all genotypes, and one therapeutic strategy is to improve the function of dystrophic muscles, in order to delay loss of ambulation or upper limb motor capacity [3, 4]. Among the biological pathways capable of impacting dystrophic muscle, TGF-β-family ligands, including myostatin and activin A, may have the potential to modify disease [5–7]. Both ligands employ activin receptors to initiate intracellular signaling that controls the proliferative state of cells. Myostatin, also known as GDF-8, is predominantly expressed in skeletal muscle and has been demonstrated to be a negative regulator of skeletal muscle proliferation [8, 9]. In animal models, genetic manipulation of myostatin through gene knockout results in muscle-specific hypertrophy and improved muscle function in the *mdx* mouse model of DMD [8, 10, 11].

Activins are a homologous family of systemically expressed, homodimeric and heterodimeric growth factors that were discovered via their primary role in regulating gonadotropin release from the anterior pituitary [12, 13]. In addition to the reproductive axis, activins have been shown to regulate hepatocyte proliferation and be critical for embryonic neurodevelopment [14–16]. Elevated levels of activin A are found in tissues and serum from a variety of inflammatory conditions [17, 18]. In dystrophic muscle, the ability of activin A to influence macrophage differentiation and control myofibroblast production suggests that reducing activin A exposure may decelerate the process of muscle degeneration and fibrosis that is a hallmark of DMD pathology [19–21]. In addition to possible anti-inflammatory effects, lowering of systemic activin A concentrations can impart muscle hypertrophy that appears to be independent of myostatin pathway signaling [22, 23].

As circulating ligands for extracellular receptors, myostatin and activin A are attractive targets for pharmaceutical intervention by functional antagonists. Several biopharmaceutical agents capable of antagonizing binding of myostatin or myostatin and activin A have entered clinical development for muscle-wasting diseases or muscular dystrophies [24–31]. However, the published clinical efficacy in sarcopenia or DMD for these agents has been modest. Possible explanations include a limitation on the pharmacodynamic benefit achievable with pure anti-myostatin agents [32, 33] or a limited therapeutic window such as seen with the recombinant activin type IIB receptor ACE-031 [26].

The endogenous ligand-binding partner for myostatin and activin A is follistatin, a systemically-expressed, circulating glycoprotein, initially discovered through its effects on regulating secretion of follicle-stimulating hormone [34]. Transgenic mouse studies have shown that dual antagonism of myostatin and activin A by follistatin overexpression has the capacity to produce larger muscle mass increases than antagonism of myostatin alone [35, 36]. In the *mdx* mouse model, follistatin overexpression either through transgenes or viral vector delivery resulted in increased muscle mass and improved dystrophic pathology [37–39]. Delivery of the follistatin gene to dystrophic muscle by local intramuscular injection of an adeno-associated virus is currently in clinical trials for DMD [40–43].

One challenge to the development of a systemically acting, follistatin-based biopharmaceutical agent is overcoming its rapid systemic clearance rate. Follistatin is a potent binder of heparin and heparan sulfate-containing proteoglycans, which sequester the protein to the vascular endothelium [44–47]. A previous publication [48] described our efforts to design a long-acting, follistatin-based molecule named FS-EEE-hFc and described its in vitro binding and pharmacokinetic properties. FS-EEE-hFc contains three glutamate mutations that reduce heparin binding and result in larger systemic exposure than native follistatin, while retaining potent binding of myostatin and activin A. Here, we describe the pharmacodynamic properties of FS-EEE-hFc, and its mouse surrogate FS-EEE-mFc, to produce muscle mass increases in wild-type mice and to improve dystrophic muscle pathology in the *mdx* model. In addition, in the *mdx* model, we compare the effects of treatment with FS-EEE-mFc to treatment with a myostatin-specific antibody and show that improvement in muscle function and dystrophic pathology is greater with FS-EEE-mFc compared to the anti-myostatin antibody.

## Methods

### Antibodies and protein reagents

FS-EEE-hFc was prepared and purified as described previously [48]. FS-EEE-mFc was prepared by cloning FS315 cDNA (Clontech, Mountain View, CA) in frame to Clontech mouse spleen cDNA for murine IgG1 Fc. Three amino acids, K76, K81, and K82, were mutagenized to glutamic acid by replacement with a mutated DNA sequence, which was synthesized by ATUM (Newark, CA). The plasmid was transfected into CHO GS cells (Sigma, St. Louis, MO) by electroporation. The cells were cultured in EX-Cell CD CHO Fusion media (Sigma) under standard condition (5% CO_2_, 37 °C) and selected for 10 days to generate stable pools. FS-EEE-mFc protein stably secreted in cell culture supernatant was captured onto a HiTrap MabSelect SuRe column (GE Healthcare Bio-Science, Piscataway, NJ) under high salt condition (2.5 M NaCl) and eluted using a step-gradient of 100 mM sodium citrate, pH 2.5. The protein was neutralized to pH 7.0 by adding 1 M Tris buffer, pH 9.0. Impurities were further removed using a Superdex 200 26/600 column (GE Healthcare Bio-Science). Purity of the final protein was > 95% as analyzed by size-exclusion chromatography [49] analysis. Recombinant mouse activin RIIB Fc chimera protein was purchased from R&D Systems (Minneapolis, MN). The anti-myostatin antibody was constructed using sequences from OGD1.0.0 [50] within a mouse IGKV6 light chain, a mouse IGHV5 heavy variable chain, and a mouse IGHG1 heavy constant chain. The monoclonal antibody was transiently expressed in CHOZN-EBNA cells (Sigma). Protein expression was carried out as a fed-batch culture in Fed-Batch Media (SAFC Biosciences, Lenexa, KS) over 14 days with daily feeds of 3% *v*/v SAFC Advanced Feed 1. Purification was achieved by capturing the conditioned media on MabSelect SuRe resin, followed by Q and SP column steps. The resulting purified protein was dialyzed into PBS pH 7.4 and had > 99% purity by SEC.

### Animals

Male C57BL/6J mice, aged 8–9 weeks, were obtained from Jackson Laboratories (Bar Harbor, ME) and acclimated for 1 week prior to start of study. For all studies, mice were housed in groups of up to five per cage in a colony room under a 12-h light-dark cycle, targeted humidity (50% ± 20%) and temperature (22 °C ± 3 °C). Rodent diet (LabDiet-5001, St Louis, MO) and water (Lexington, MA municipal water purified by reverse osmosis) were available ad libitum for the duration of the experiment. Male *mdx* mice (C57BL/10ScSn-Dmd<*mdx*>/J) and C57BL/10ScSnJ were sourced from Jackson Laboratories and bred to obtain animals for the study. Mice were balanced across treatment groups using body weight at 3 weeks (± 3 days) of age for the unexercised study and 5 weeks for the exercised study. To prevent litter effects, *mdx* animals from the same litter were distributed across groups. During the course of the study, 12 h/12 h light/dark cycles (unexercised), 13 h/11 h light/dark cycles (exercised), and a room temperature of 20 to 23 °C were maintained with a relative humidity maintained around 50%. Food and water were provided ad libitum for the duration of the study. All assessments were performed during the animals’ light cycle phase with dose groups randomized and test article identifications blinded. For treadmill exercise, mice were placed on a treadmill (Columbus Instruments, Columbus, OH) set at a 0° incline one at a time (up to five mice per lane) in a lane and run for 30 min at a maximum speed of 12 m per minute, twice a week.

### Dosing and blood sampling

IV administration of the FS-EEE-mFc and FS-EEE-hFc was performed using tail vein injection. For subcutaneous (SC) injections, a ½ cc insulin syringe containing a 27 gauge needle was filled with test articles diluted in PBS to 1–2 mg/mL, and animals injected in the interscapular area. Blood was collected via submandibular bleeds prior to dosing at timepoints indicated. A terminal bleed was also conducted 24 h post last dose. Whole blood was collected in Becton Dickinson (Franklin Lakes, NJ) Microtainer® serum separator tubes and processed as directed.

### Grip strength

Grip strength was measured on a Chatillion DFE II Force Gauge (San Diego Instruments, San Diego, CA) for the unexercised study and a Grip Strength Meter (Columbus Instruments, Columbus, OH) for the exercised study. Animals were tested in five consecutive trials. At an angle of 0°, in one continuous, fluid motion, animals’ forelimb grip strengths were assessed. In the unexercised study, grip strength was measured on one day after 11.5 weeks of dosing. In the exercised study, animals were acclimated to the grip strength apparatus by 5 days of unrecorded testing during week 9 followed by 5 days of recorded testing during week 10.

### Exhaustion assay

In the exercised study, the exhaustion assay was performed using a treadmill with four lanes and a running plane of 0°. Mice were randomized such that each mouse running in one apparatus was from a different treatment group. After a 5 min at 5 m/min acclimation period, the speed of the treadmill was increased by 1 m/min every minute until the mice were exhausted, as defined by inability to continue running for 20 consecutive seconds despite repeated gentle nudges. The exhaustion assay was performed on each mouse three times with a day of rest in between testing days and the average distance of running (m) was measured.

### Ex vivo force muscle contractions

Contractile properties were measured ex vivo on the EDL muscle at the end of the study. Mice were anesthetized, and the EDL muscle of the right hindlimb was removed from each mouse and immersed in an oxygenated bath (95% O_2_, 5% CO_2_) containing Ringer’s solution (pH 7.4) at 25 °C. The muscle was flanked by two electrodes, and using non-fatiguing twitches, the muscle was adjusted to the optimal length for force generation. The force frequency curve was generated using 30, 80, 100, 150, 180, 200, and 250 Hz. The maximal force was measured with the muscle held at optimal length. The muscles were stimulated with electrodes to elicit tetanic contractions that were separated by 2-min rest intervals. With each subsequent tetanus, the stimulation frequency was increased in steps of 20, 30, or 50 Hz until the force reached a plateau which usually occurred around 250 Hz. That plateau was considered the maximum force [51] generated by the muscle. The cross-sectional area (CSA) of the muscle calculated using the formula below was measured based on muscle mass (value obtained using calibrated analytic scale), optimal fiber length (using a vernier caliper), and tissue density. Muscle-specific force (kN/m^2^) was calculated based on the cross-sectional area of the muscle calculated as follows. CSA = muscle mass/(optimal length of the EDL × 0.45 × 1.056). The fiber to muscle length ratio is 0.45. The density of muscle is 1.056. Optimal length was measured when the EDL produced the maximal tetanus force**.**

### Serum biomarkers

Creatine kinase was measured in serum samples using a Cobas C311 Clinical Chemistry Analyzer (Roche). Samples were diluted 1:16 in RODI water and analyzed using a Creatine Kinase test kit (Roche, Cat# 4524977190). Samples were analyzed along with the appropriate assay calibrator (Roche C.F.A.S, Cat# 10759350360) and controls (Roche Precinorm U Plus, cat#1214935160, and Precipath U Plus, cat#12149443160). Muscle injury markers sTn1 and cTn1 were measured in serum samples using Muscle Injury panel 3 (MSD, Rockville, MD #K15186C-5) according to the manufacturer’s instructions. Mouse serum samples were prepared by diluting the serum eightfold in Diluent 33 with EDTA and DTT added. Twenty-five microliters per well of samples and standards were loaded onto the MSD plates and incubated at 25 °C for 2 h with vigorous shaking (300–1000 rpm). After the incubation plates were washed three times with 300 uL/well of PBST, twenty-five microliters per well of sulfo-tagged detection antibody solution was added and plates were incubated for an additional 2 h with vigorous shaking at 25 °C. Plates were washed three times with 300 uL/well of PBST. One hundred fifty microliters of 1× Read Buffer T was added to each well and plates were read with a MSD SECTOR imager.

### FS-EEE-mFc bioanalysis

Mouse serum concentrations of FS-EEE-mFc were determined using an electro-chemiluminescent immunoassay. Meso Scale Discovery (MSD, Rockville, MD) standard plates were coated with 2.5 μg/mL goat anti-human Follistatin Ab (R&D Systems, AF669) in PBS, 50 μL/well. After overnight incubation at 4 °C, plates were washed three times with wash buffer [1× Dulbecco’s PBS (Gibco #14190-136 or equivalent) + 0.02% Tween 20] and then blocked with 150 μL/well 0.5% Blocker B/2.5% Blocker A (MSD) and incubated at 25 °C for 1 h with shaking. After washing three times with wash buffer, samples and a standard curve of FS-EEE-mFc from 12.5 to 0.098 ng/mL were added, 25 μL/well. Samples, controls, and standards were diluted in 10% mouse serum matrix if required dilutions were beyond 1:10. After washing three times with wash buffer, a 1:8000 dilution of rabbit anti-mouse IgG1 (Abcam, Cambridge, MA, ab125913), diluted in 0.5% Blocker B/2.5% Blocker A, was added, 25 μL/well. After incubation at 25 °C for 1 h with shaking, and then washing three times with wash buffer, 1 μg/mL Sulfo-tag labeled goat anti-rabbit Ab (MSD, R32AB-1) was added, 25 μL/well. After incubation at 25 °C for 1 h with shaking, and washing three times with wash buffer, 1× Read Buffer T (MSD) was added, 150 μL/well. Plates were read with a MSD SECTOR imager and concentrations determined relative to the standard curve, adjusted for dilutions.

### Anti-MST mAb bioanalysis

Mouse serum concentrations of Anti-MST mAb were determined using an electro-chemiluminescent immunoassay. Meso Scale Discovery (MSD, Rockville, MD) standard plates were coated with 10 ng/well recombinant human/mouse/rat GDF-8/Myostatin (R&D Systems, 788-G8-010) in PBS, 30 μL/well. After overnight incubation at 4 °C, plates were washed three times with wash buffer [1× Dulbecco’s PBS (Gibco #14190-136 or equivalent) + 0.02% Tween 20] and then blocked with 150 μL/well 2.5% BSA + 0.05% Casein + 0.05% Tween 20 and incubated at 25 °C for 1 h with shaking. After washing three times with wash buffer, samples, controls, and a standard curve of Anti-MST mAb from 125 to 0.977 ng/mL were added to the plate, 25 μL/well. Samples, controls, and standards were diluted in 1% mouse serum matrix if required dilutions were beyond 1:100. After incubation at 25 °C for 1 h with shaking, and then washing three times with wash buffer, 1 μg/mL Sulfo-tag labeled goat anti-mouse Ab (MSD, R32AC-1) was added, 25 μL/well. After incubation at 25 °C for 1 h with shaking, and washing three times with wash buffer, 1× Read Buffer T (MSD) was added, 150 μL/well. Plates were read with a MSD SECTOR imager and concentrations determined relative to the standard curve, adjusted for dilutions.

### Tissue collection

Animals were anesthetized with sodium pentobarbital and perfused with saline. For the diaphragm, right side was immediately placed into RNALater® solution. Once the sample was submerged, clean blades were used to cut the sample into smaller pieces and then stored at 4 °C for 24 h. After 24 h, remaining RNALater® Solution was removed and tissue samples were then immediately placed on dry ice, prior to storage at − 80 °C. The left side of the diaphragm with attached rib cage was dropped fixed in 10% neutral buffered formalin (NBF) for 72 h and then transferred to 70% alcohol for storage at 4 °C. Quadriceps tissue was handled as above with right side stored in RNALater® solution and left side fixed in 10% NBF.

### Histological analysis

The fixed tissues were processed for paraffin embedding, and 5-μm sections were prepared. Hematoxylin and eosin (H&E) staining was performed following standard procedure for a Leica stainer. The stained slides were scanned with an Aperio ScanScope AT2 scanner. The digital slides were viewed, and the area of rectus femoris muscle from quadriceps was measured with ImageScope.

### Myofiber size measurement

Slides were de-paraffinized in xylene and rehydrated through alcohol to water to PBS. Twenty microliters of Oregon Green® 488 WGA (ThermoFisher, Waltham, MA, W6748, 1:250 dilution) was applied to each slide and incubated overnight at 4 °C, rinsed in PBS and mounted using an anti-fading mounting medium with 4′,6-diamidino-2-phenylindole (DAPI) for nuclei counterstaining. Stained quadriceps sections were scanned with Aperio FL scanner. 20× photos were taken from the scanned digital image, three photos from each muscle in the similar area. The mean myofiber diameter was measured with Image-Pro Plus software.

### Immunohistochemistry

All immunohistochemistry staining was performed in 5-μm paraffin sections with BondRX Stainer. Briefly, the primary antibodies including rabbit anti-mouse IgG from Bond Polymer Refine kit (Leica Biosystems, Buffalo Grove, IL, ready to use), rabbit anti-CD68 antibody (Abcam, ab125212, 1:500), and rabbit anti-Collagen1 antibody (BosterBio, Pleasanton, CA, PA2140-2, 1:1000) were used for detection of necrosis, inflammation, and fibrosis, respectively. Bond Polymer Refine kit (Leica, Cat No: DS9800) was applied as the detection system. The positive cells were identified as brown in color and nuclei were stained blue. The stained slides were scanned with Aperio ScanScope AT2 scanner. The whole digital slides were viewed and analyzed by ImageScope. The positive pixel count algorithm was selected and adjusted to cover each individual positive staining for analysis. The data was presented as positivity which was obtained from the following formula: positivity (%) = positive area (pixels)/total analyzed area (pixels) × 100%.

### qPCR

Mouse muscle tissues were treated with RNALater® and stored at − 80 °C. RNA extraction was performed by using miRNeasy Mini Kit (Qiagen, Germantown, MD) according to the manufacturer’s instructions. Briefly, the entire piece of tissue was homogenized in QIAzol Lysis Reagent using a handheld rotor-stator homogenizer TissueRuptor (Qiagen) attached with a disposable probe (Qiagen) for 60 s. Seven hundred microliters of tissue lysate was mixed vigorously with 140 μL chloroform for 15 s. The upper aqueous phase containing RNA was separated by centrifugation and was mixed with 1.5 volumes of 100% ethanol for RNA binding using the RNeasy mini column. The on-column DNase digestion was performed using RNase-Free DNase Set (Qiagen) according to instructions. After washing steps, RNA was eluted with RNase-free water into 1.5 mL RNase-free, individually wrapped/sterilized microcentrifuge tubes (ThermoFisher). RNA yield was determined by a Nano-drop spectrophotometer (ThermoFisher). TaqMan primer/probe sets were obtained from ThermoFisher for mouse Acta2 (Mm00725412_s1), CD68 (Mm03047343_m1), Col1a1 (Mm00801666_g1), Cthrc1 (Mm01163611_m1), Hprt (Mm01545399_m1), Lox (Mm01265612_m1), spp1 (Mm00436767_m1), and Tgfb1 (Mm01178820_m1). RT-PCR reactions (10 μL) in 384-well plates contained 50 ng RNA and Taqman master mixtures (RNA-to-Ct 1-step kit, ThermoFisher) as directed by manufacturer. RNA samples were tested in quadruplicate and each plate contained an internal calibrator sample randomly selected from the vehicle control animal group. PCR was performed in a LightCycler 480 (Roche) and relative quantitation values calculated using Hprt as the reference gene. Samples with standard deviations among replicates of > 0.2 were repeated.

### Statistical analysis

All data was analyzed with Prism 7 (GraphPad Software). Error bars on plots represent SEM. *P* values for drug treatment groups compared to vehicle control were generated by one-way ANOVA with Dunnett’s correction for multiple comparisons.

## Results

### Engineered follistatin and systemic delivery results in muscle hypertrophy in wild-type mice

**Fig. 1:**
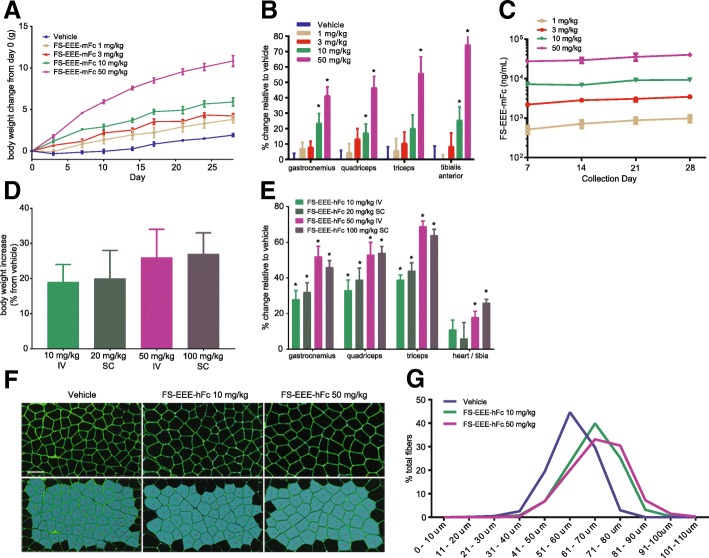
Body weights, muscle weights, serum drug concentrations, and morphometric analysis from a 4-week C57BL/6 mouse study. **a** Body weights from dosing of FS-EEE-mFc. **b** Muscle weights from dosing of FS-EEE-mFc. **c** Drug concentrations of FS-EEE-mFc from serum samples taken immediately prior to dosing. **d** Body weight changes at day 28 from dosing of FS-EEE-hFc. **e** Muscle weights from dosing of FS-EEE-hFc. **f** Quadriceps morphometric analysis by Oregon Green® 488 WGA staining of quadriceps. **g** Histogram of myofiber diameters. *p < 0.05 compared to vehicle-dosed group as described in the “Methods” section

Two engineered follistatin molecules were employed in studies with wild-type C57BL/6 mice and a 4-week period of dosing. In one study, FS-EEE-mFc was dosed twice weekly intravenously from 1 to 50 mg/kg. Upon FS-EEE-mFc dosing, body weights increased in a dose-dependent manner (Fig. 1a), which was linked to skeletal muscle mass increases (Fig. 1b). Serum concentrations of FS-EEE-mFc were measured using an electro-chemiluminescent immunoassay and as shown in Fig. 1c, trough levels of FS-EEE-mFc were dose proportional from 1 to 50 mg/kg. The FS-EEE-hFc molecule was evaluated following twice weekly subcutaneous and intravenous administration. FS-EEE-hFc dosed 10 mg/kg IV or 20 mg/kg SC resulted in similar effects on body weight at 20% increase, and individual muscle mass increases ranged from 28 to 44%. FS-EEE-hFc-dosed 50 mg/kg IV or 100 mg/kg SC resulted in similar effects on body weight at 26% increase and individual muscle mass increases ranged from 46 to 69% (Fig. 1d, e). Heart weights were normalized to tibia length, and an increase in heart/tibia ratio was seen at the higher doses of FS-EEE-hFc. Quadriceps tissue samples were examined for morphological differences from vehicle treatment. Using immunofluorescence microscopy, larger myofiber sizes were observed upon FS-EEE-hFc dosing (Fig. 1f), compared to vehicle-dosed animals. Average myofiber diameter was increased compared to vehicle for FS-EEE-hFc at both dose levels (Fig. 1g).

### In *mdx* mice follistatin treatment results in muscle hypertrophy and improvement in muscle function

**Fig. 2:**
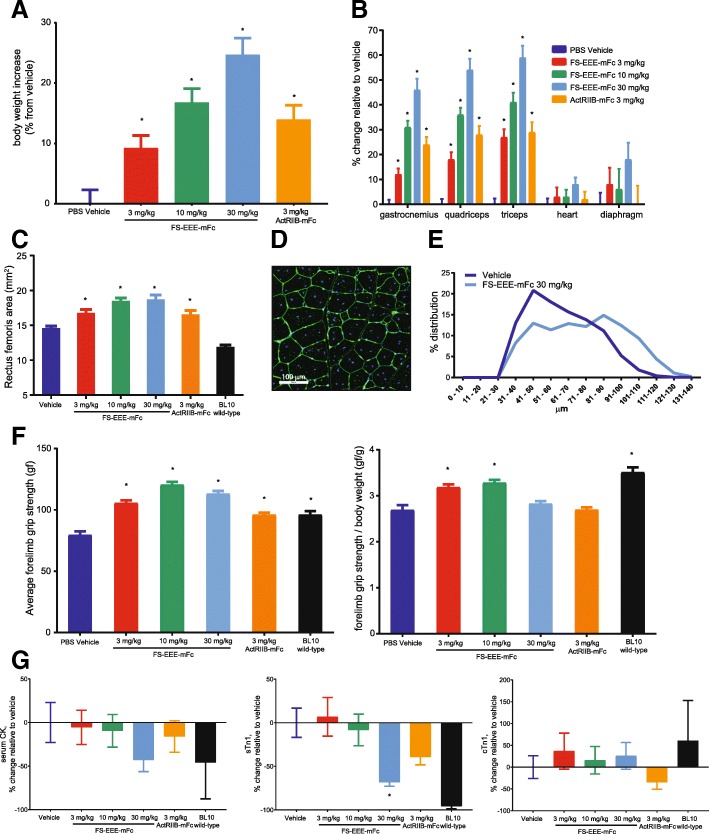
Body weights, muscle weights, muscle fiber size, grip strength, and serum biomarkers from a 12-week unexercised *mdx* study. **a** Body weights. **b** Muscle weights. **c** Quadriceps rectus femoris area. **d** Oregon Green® 488 WGA staining of quadriceps, example from the vehicle group. **e** Quadriceps morphometric analysis histogram of myofiber diameter size distribution. **f** Forelimb grip strength: (left) absolute and (right) normalized to body weight. **g** Serum biomarkers: (left) creatine kinase, (middle) skeletal troponin 1, (right) cardiac troponin 1. *p < 0.05 compared to *mdx* vehicle-dosed group as described in the “Methods” section

To evaluate effects on dystrophic muscle, the follistatin FS-EEE-mFc molecule was dosed to 3-week-old *mdx* mice twice weekly for 12 weeks by subcutaneous administration. Three doses for FS-EEE-mFc were selected ranging from 3 to 30 mg/kg and compared to an Fc fusion of the recombinant activin type IIB receptor (ActRIIB-mFc) dosed at 3 mg/kg, also subcutaneous twice weekly. Mice were not subjected to regular exercise and were assessed for forelimb grip strength at week 10 of the study. As seen in Fig. 2a, body weights increased for FS-EEE-mFc across doses and ranged from 9 to 25% compared to the ActRIIB-mFc at 14%. Skeletal limb muscle increases ranged from 12 to 27% with 3 mg/kg FS-EEE-mFc to 46 to 59% with 30 mg/kg FS-EEE-mFc (Fig. 2b). The increases in weights of hearts and diaphragms were smaller than limb muscles and not significantly different from PBS vehicle treatment. From the quadriceps, the area of the rectus femoris was quantified and significant increases were observed for all drug-treated groups (Fig. 2c). In addition, myofiber sizes were quantified and average myofiber diameter increased upon FS-EEE-mFc treatment compared to the vehicle control (Fig. 2d, e).

All doses of FS-EEE-mFc restored absolute forelimb grip strength to a level greater than that of C57BL/10 wild-type mice, with maximal effect at 10 mg/kg FS-EEE-mFc (Fig. 2f). When normalized to body weight, both 3 and 10 mg/kg FS-EEE-mFc increased grip strength to a level greater than the *mdx* vehicle control and similar to the wild-type level. Effects on circulating markers of muscle damage were measured. Serum creatine kinase activity was highly variable and the highest dose of FS-EEE-mFc resulted in the largest reduction compared to vehicle treatment (Fig. 2g). Skeletal troponin I levels were reduced at the highest FS-EEE-mFc dose but cardiac troponin I levels remained unchanged, in agreement with the greater observed hypertrophy in limb muscles compared to heart.

### Quadriceps and diaphragm pathology are improved upon engineered follistatin treatment

**Fig. 3:**
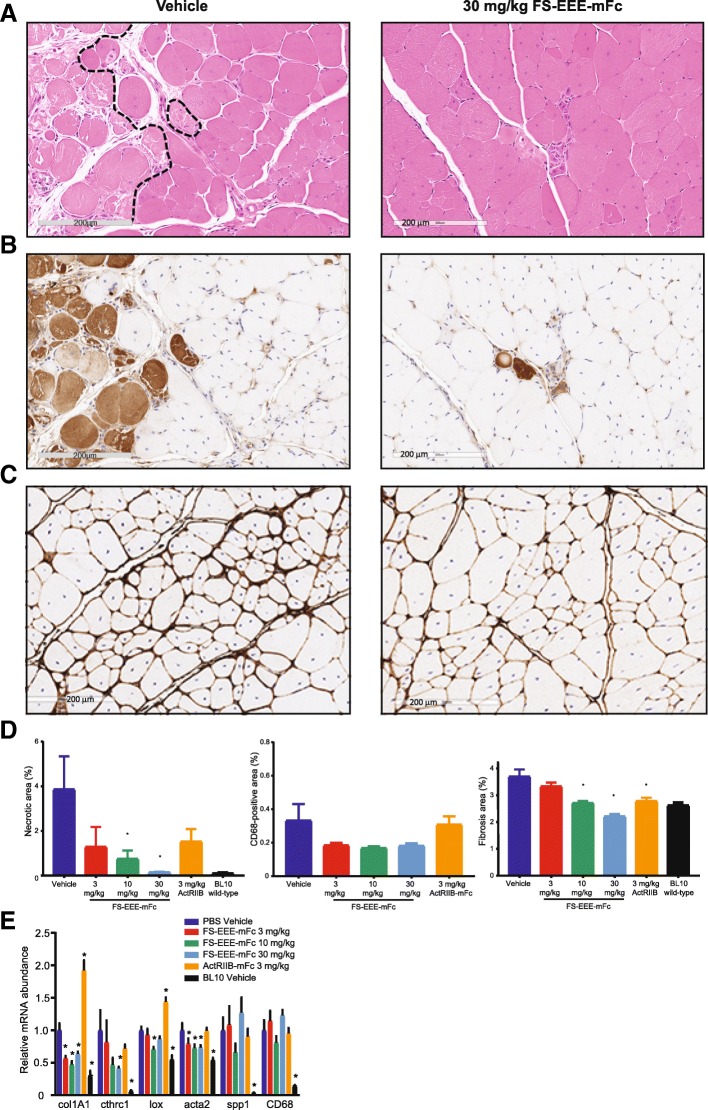
Histological staining and qPCR analysis of *mdx* quadriceps. **a** Representative images of hematoxylin and eosin staining depicting areas of heterogeneous necrosis from the vehicle control and 30 mg/kg FS-EEE-mFc. Dashed lines on the vehicle image depict manually defined boundaries of necrotic areas. **b** Representative images of mouse IgG-positive staining depicting areas of heterogeneous necrosis from the vehicle control (left) and 30 mg/kg FS-EEE-mFc (right). **c** Representative images of collagen I staining from vehicle control (left) and 30 mg/kg FS-EEE-mFc (right). **d** Total slide image analysis of IgG-positive staining for necrosis (left), CD68-positive staining for macrophage infiltration (center), and collagen I-positive staining for fibrosis (right). **e** qPCR of fibrosis and inflammation markers. *p < 0.05 compared to *mdx* vehicle-dosed group as described in the “Methods” section

To evaluate effects upon dystrophic pathology, both quadriceps and diaphragm tissues were analyzed by immunohistochemistry whole-slide analysis for markers of tissue necrosis, inflammation, and fibrosis. As a marker for necrosis, we developed an IHC method to detect endogenous mouse IgG with anti-mouse IgG, taking advantage of necrotic area IgG accumulation, which binds to histidine-rich glycoprotein (HGP) to form HGP-IgG complexes that facilitate necrotic cell clearance [52, 53]. In *mdx* muscle, as assessed by comparison to hematoxylin and eosin staining, mouse IgG IHC accurately labeled necrotic cells, although areas of necrosis were variable in tissue sections (Fig. 3a, b). In order to best account for variability, entire slide images were analyzed for quantification and cohort animal numbers were high for each group (*n* = 15). In the whole-slide analysis of quadriceps, statistically significant reduction in necrotic tissue area was achieved at the 10 and 30-mg/kg doses of FS-EEE-mFc and not for the 3-mg/kg dose of ActRIIB-mFc (Fig. 3d). Similar to the finding for areas of necrosis, staining for CD68, a marker for pro-inflammatory M1-type macrophages, revealed patchy areas of positive staining. Due to the low overall level of detectable macrophage infiltration, when entire slide images were quantified for CD68-positive area, drug treatment effects did not reach significance (Fig. 3d).

Collagen I detection was able to identify 4% positive staining area in the vehicle control that was significantly reduced in both 10 and 30 mg/kg of FS-EEE-mFc and the 3 mg/kg of ActRIIB (Fig. 3c, d). The overall pattern of histopathological analysis in quadriceps is consistent with hypertrophy of pre-existing, centronucleated, and regenerating myofibers. Expansion of regenerating cells resulted in reduced degeneration, and with less damaged, necrotic tissue to drive collagen deposition in the extracellular matrix, FS-EEE-mFc reduced fibrosis.

To corroborate the histopathology results, gene markers for fibrosis were measured from homogenates of quadriceps tissue. As seen in Fig. 3e, all three doses of FS-EEE-mFc-reduced expression of genes related to deposition and cross-linking of collagen, col1A1, lox, cthrc1, and acta2. Transcript levels were not reduced for CD68 or spp1, which encodes for osteopontin, a highly expressed extracellular protein in dystrophic muscle that has genetic linkage to fibrosis development in the *mdx* model [54] and DMD disease severity [55]. In mRNA analysis, the ActRIIB-mFc group displayed no reduction and in some cases increased levels of gene markers for fibrosis and inflammation.

**Fig. 4:**
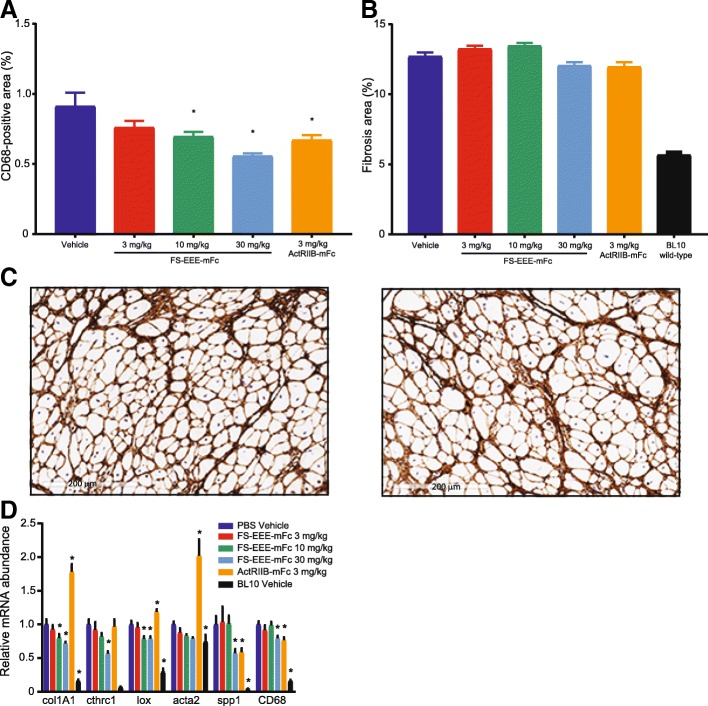
Immunohistochemistry staining and qPCR analysis of *mdx* diaphragm. **a** Image analysis of CD68-positive staining. **b** Image analysis of collagen I-positive staining. **c** Representative magnified images of collagen I-stained diaphragm: (left) vehicle control and (right) 30 mg/kg FS-EEE-mFc. **d** qPCR of inflammation and fibrosis markers. *p < 0.05 compared to *mdx* vehicle-dosed group as described in the “Methods” section

In diaphragm tissue, the baseline level of CD68-positive macrophage infiltration was higher than in quadriceps, and reductions were observed at 10 mg/kg and 30 mg/kg of FS-EEE-mFc and also 3 mg/kg of ActRIIB-mFc (Fig. 4a). Collagen I immunohistochemistry revealed a higher level of fibrosis in diaphragm compared to quadriceps, at 12% vs 4% for the vehicle control in both muscles (Fig. 4b vs Fig. 3d). Unlike quadriceps, in diaphragm collagen I, content was not significantly altered upon drug treatment. Quantitative RT-PCR of genes involved in fibrosis and inflammation showed reduction in RNA expression at the 10 and 30-mg/kg doses of FS-EEE-mFc (Fig. 4d). Similar to the quadriceps, the ActRIIB-mFc group’s gene transcript responses were increased for markers of fibrosis.

### Follistatin treatment of *mdx* mice results in greater improvement in muscle function and pathology than treatment with a myostatin antagonist

To compare the effects of engineered follistatin to an agent specific for myostatin antagonism, a monoclonal antibody designed to bind specifically to myostatin was prepared. The resulting antibody, containing a mouse IgG Fc, was compared to FS-EEE-mFc for ability to bind the ligands myostatin and activin A using a surface plasmon resonance method [48]. Both FS-EEE-mFc and the anti-MST antibody bound myostatin tightly, with *K*_D_ values of 7.5 and 15 pM, respectively, whereas for activin A FS-EEE-mFc displayed a *K*_D_ of 6.1 pM and the anti-MST antibody displayed no detectable binding.

Next, both molecules were compared for effects on dystrophic muscle in mice. In this study, *mdx* mice aged 5 weeks and subjected to a regular exercise regimen were dosed for 12 weeks by subcutaneous administration. Two doses of each molecule were selected, 3 and 30 mg/kg; however, based on a longer predicted half-life for the antibody, frequency of FS-EEE-mFc dosing was set to twice weekly compared to once weekly for the anti-myostatin antibody.

**Fig. 5:**
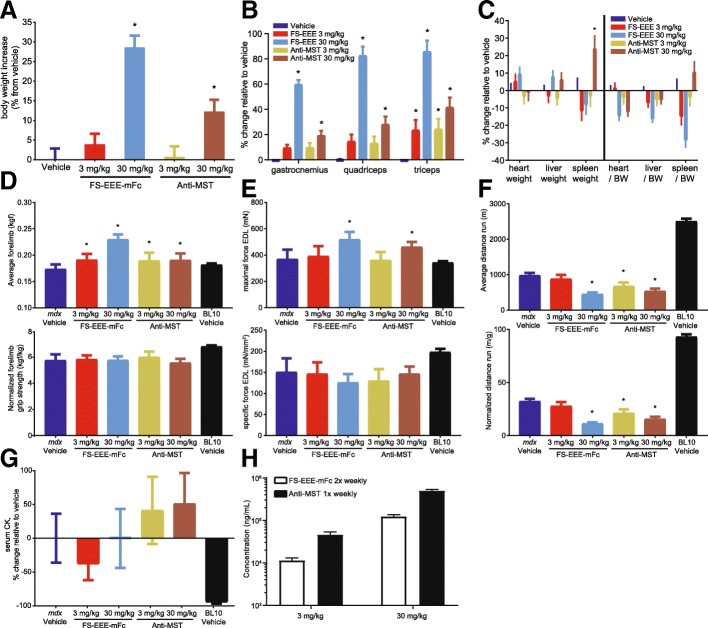
Body weights, tissue weights, functional measurements, behavioral measurements, and serum analyses from a 12-week exercised *mdx* study. **a** Body weights. **b** Muscle weights. **c** Organ weights. **d** Forelimb grip strength (top) and normalized to body weight (bottom). **e** Ex vivo force of EDL muscle (top) and normalized to cross-sectional area (bottom). **f** Forced treadmilling distance (top) and normalized to body weight (bottom). **g** Serum creatine kinase. **h**. Serum drug concentrations sampled at day 56. *p < 0.05 compared to *mdx* vehicle-dosed group as described in the “Methods” section

Body weight and muscle mass increases were seen with both doses of both agents (Fig. 5a, b). The magnitude of body weight and muscle mass increase was greater at the 30-mg/kg dose of FS-EEE-mFc compared to 30 mg/kg of the anti-MST antibody. At the 3-mg/kg dose, body weight and muscle mass increases were greatly reduced compared to 30 mg/kg, and the magnitudes of effects for both agents were comparable. Heart, liver, and spleen weights, both absolute and normalized to body weight, were not altered, except for an increase in spleen weight with the higher dose of the anti-MST antibody (Fig. 5c).

Functional and behavioral measurements were recorded following animal acclimatization to instrumentation as recommended for *mdx* studies [56]. In forelimb grip strength, both doses of both agents resulted in increases above vehicle treatment (Fig. 5d). The 30-mg/kg dose of FS-EEE-mFc generated a larger increase than 30 mg/kg of the anti-MST antibody. After normalization to body weight, the grip strength increases were not distinguished from vehicle treatment. Isolated tetanic force of the EDL muscle was measured at the end of the study (Fig. 5e). Only the 30-mg/kg doses of both agents resulted in increased tetanic force, and the FS-EEE-mFc increase was greater than the anti-MST antibody increase. When normalized to EDL cross-sectional area, specific force was not distinguishable from the *mdx* vehicle-dosed group. Forced treadmilling was examined and reductions in running distance were seen for the 30-mg/kg group of FS-EEE-mFc as well as both doses of the anti-MST antibody (Fig. 5f). When normalized to body weight, these reductions compared to vehicle were maintained.

Serum CK analysis displayed a high level of variability within groups that precluded appearance of significant differences among groups (Fig. 5g). Serum was also analyzed for drug concentrations during week 8 of the study. As seen in Fig. 5h, dose proportionality was evident for both agents, with the 30-mg/kg doses resulting in approximately tenfold higher concentrations than the 3-mg/kg doses. Even though it was dosed less frequently, the anti-MST antibody concentrations were about fourfold higher than FS-EEE-mFc. Comparing the serum concentrations to hypertrophic effect, at the 3-mg/kg dose, a fourfold lower serum concentration of FS-EEE-mFc than the anti-MST antibody generated similar muscle mass effects. This trend was more pronounced at the 30-mg/kg dose, where greater muscle mass, forelimb grip strength, and EDL tetanic force increases were seen for the FS-EEE-mFc compared to the anti-MST-antibody, despite fourfold less of the FS-EEE-mFc drug in circulation.

Quadriceps and diaphragm tissues were analyzed by immunohistochemistry and qPCR for changes in dystrophic pathology. Compared to the unexercised study, in the exercised study, similar background levels of quadriceps and diaphragm muscle damage were observed in the vehicle control groups. This was surprising given reports documenting worsening limb muscle necrosis and diaphragm fibrosis in exercised vs unexercised *mdx* [57, 58]. One possible explanation may be the young animal ages in our studies (starting at 3 weeks in unexercised, 5 weeks in exercised), which meant that mice were dosed through a period of high limb muscle regeneration [59]. Another factor was our choice of level treadmilling instead of downhill treadmilling.

**Fig. 6:**
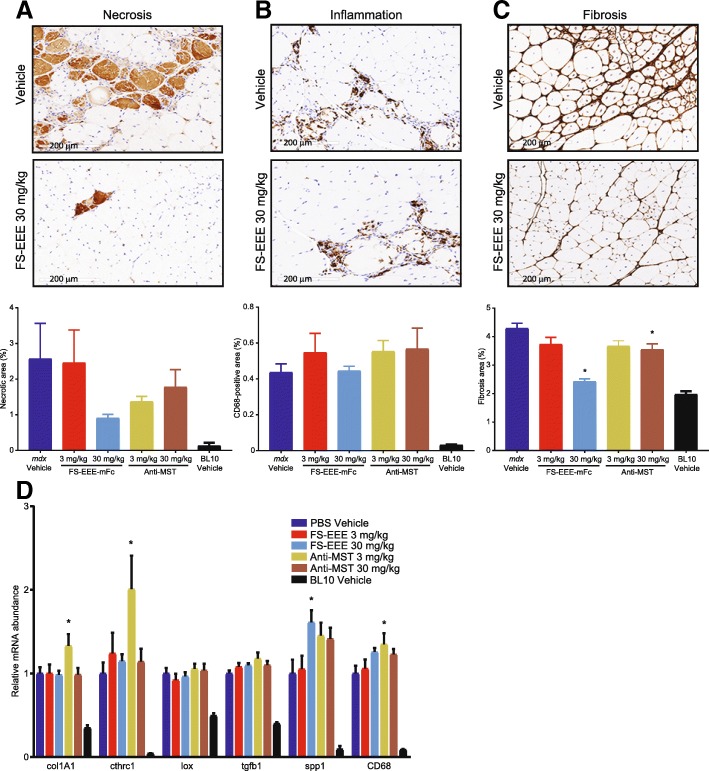
Quadriceps tissue analysis from a 12-week exercised *mdx* study. (A-C) Representative images from the (top) vehicle control and (middle) 30 mg/kg FS-EEE-mFc and (bottom) total slide image analysis for **a** mouse IgG-positive staining for necrosis, **b** CD68-positive staining for macrophage infiltration, and **c** collagen I-positive staining for fibrosis. **d** qPCR of fibrosis and inflammation markers. *p < 0.05 compared to *mdx* vehicle-dosed group as described in the “Methods” section

As seen in Fig. 6a–c, compared to vehicle treatment in the quadriceps, the 3-mg/kg dose of both agents produced small reductions in muscle necrosis and fibrosis. At 30 mg/kg, large reductions in necrosis and fibrosis were seen for FS-EEE-mFc compared to small reductions for the anti-MST antibody. CD68-positive macrophage staining did not distinguish treatment groups from vehicle control, which may have been limited by the low levels of baseline staining of this marker. mRNA analysis of the contralateral quadriceps muscles for markers of fibrosis and inflammation is shown in Fig. 6d. Here, reductions in transcript levels were not observed for any agent or dose, and in fact, several markers displayed slightly increased levels for the low dose anti-MST antibody (col1A1, cthrc1, CD68) and the high dose of FS-EEE-mFc (CD68). For the fibrosis gene markers, one possible explanation for the difference between the collagen I IHC and col1A1 gene expression is the age of the animals. In *mdx*, the period of severe myonecrosis in limb muscles that begins around 3 weeks of age resolves by week 8 to a state of less active damage [56, 59]. Animals were > 4 months old at termination of the study, an age beyond the window of active limb muscle degeneration that would produce the pro-inflammatory, pro-fibrotic signaling necessary to drive connective tissue deposition. As a result, the anti-fibrotic effect of FS-EEE-mFc manifested at the protein level because the gene pathways for fibrosis that were most active in the early phase of the study were quiescent at the study termination.

**Fig. 7:**
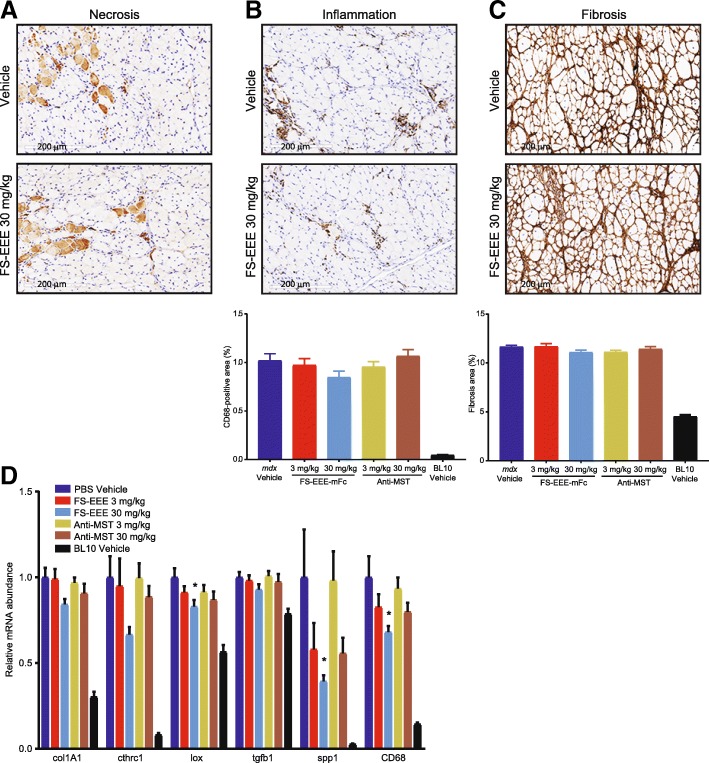
Diaphragm tissue analysis from a 12-week exercised *mdx* study. **a**–**c** Representative images from the (top) vehicle control and (middle) 30 mg/kg FS-EEE-mFc and (bottom) total slide image analysis for **a** mouse IgG-positive staining for necrosis, **b** CD68-positive staining for macrophage infiltration, and **c** collagen I-positive staining for fibrosis. Note that due to high variability in IgG staining, total image analysis not shown for **a**. **d** qPCR of fibrosis and inflammation markers. *p < 0.05 compared to *mdx* vehicle-dosed group as described in the “Methods” section

In the diaphragm, compared to the quadriceps, overall higher levels of baseline tissue damage were observed by IHC (Fig. 7a–c). Both doses of the anti-MST antibody showed no effects on CD68 or collagen I staining. For FS-EEE-mFc, qualitative reduction in CD68 macrophage infiltration was observed at 30 mg/kg; however, no significant changes were seen in collagen I staining. mRNA analysis revealed lower transcript levels of several inflammation and fibrosis markers for the 30-mg/kg dose of FS-EEE-mFc compared to vehicle treatment. The greatest reduction was seen for spp1, which encodes for osteopontin. Reducing osteopontin levels has been shown to reduce pro-inflammatory macrophage populations in favor of pro-regenerative macrophages [60]. Consistent with this pattern, along with spp1, mRNA for CD68 was also lowered at the 30-mg/kg FS-EEE-mFc dose.

## Discussion

Since the discovery of myostatin and its repressive effect on muscle proliferation, the promise of its endogenous binding partner follistatin has been recognized. Transgenic mouse studies have shown that follistatin has the potential to induce greater hypertrophy than antagonism of myostatin alone [35, 36] and the additional pharmacology of follistatin has been attributed to its sequestration of activin A in addition to myostatin [22]. More recent work has reinforced the notion that enhanced muscle hypertrophy is achievable with dual antagonism, as evidenced by co-administration of individual activin A and myostatin antagonists alone and in combination, either through AAV vector expression [61] or as monoclonal antibodies [62].

Having engineered a long-acting version of follistatin capable of binding activin A and myostatin [48], we sought to interrogate its hypertrophic effects in C57BL/6 mice. Using a chimeric surrogate containing the engineered human follistatin fused to a mouse Fc stem, after 4 weeks of administration, dose-dependent increases in body weights and muscle masses were observed. Larger increases were seen in limb muscles than in the heart, a result consistent with previous investigations of cardiac hypertrophy with myostatin blockade [10, 63, 64]. A plateau in limb muscle hypertrophy was not seen despite a top dose of 50 mg/kg that generated > 10^5^ ng/mL FST-EEE-mFc detectable in serum. Increased muscle size was shown to result from hypertrophic enlargement of existing myofibers and not from hyperplastic generation of new myofibers. Equivalent muscle mass responses to the mouse surrogate were demonstrated after administration of the engineered human follistatin fused to a human IgG1.

With primary pharmacology demonstrated in wild-type mice, we next sought to evaluate FST-EEE-mFc in the *mdx* disease model. A fundamental question was whether systemic follistatin administration could improve *mdx* muscle fibrosis. For comparators, because of their different selectivity for TGF-β ligands, we chose the Fc fusion of ActRIIB (more promiscuous than follistatin) and a monoclonal antibody specific for myostatin (more selective). As an antagonist of multiple TGF-β family ligands, ActRIIB-mFc would be expected to have the greatest potential to influence inflammation and fibrosis in *mdx*, and this has been shown by several investigators [65–67]. Less clear is the degree to which systemic administration of a specific myostatin antagonist can improve fibrosis in *mdx*. Testing in *mdx* has not been reported for some agents [27, 68, 69], and in another case, improvements in diaphragm fibrosis were shown to be dependent on the age of animals [64]. In a recent paper investigating mRK35, the mouse surrogate of domagrozumab, in *mdx*, no anti-fibrotic effects were reported due to a low level of fibrosis in the vehicle-dosed controls [30].

In the study comparing to ActRIIB-mFc, FS-EEE-mFc demonstrated limb muscle mass increases that translated to greater forelimb grip strength at all doses. Reductions in circulating biomarkers for muscle damage were seen for both ActRIIB-mFc and FS-EEE-mFc, and in the quadriceps and diaphragm, both ActRIIB-mFc and FS-EEE-mFc treatment produced reductions in tissue inflammation, necrosis, and fibrosis. Importantly, the combination of hypertrophy and reduced inflammation in *mdx* suggests that systemic follistatin delivery can manifest the benefits from dual antagonism of myostatin and activin A.

In the study comparing to an anti-MST antibody, both agents demonstrated body weight and limb muscle mass increases, with greater hypertrophy seen for FS-EEE-mFc despite lower circulating trough serum concentrations. Grip strength improvements were seen at both doses of both agents, but tetanic force increases were seen only at the higher doses of each molecule. Specific force was not significantly altered, which is similar to observations with anti-MST antibodies and ActRIIB-Fc [29, 67, 70]. Forced treadmilling distance was not improved for any treatment group, and decreases were seen for both anti-MST antibody doses and the higher dose of FS-EEE-mFc. Whether this was due to deficiencies in exercise capacity will require further investigations into the metabolic physiology of follistatin-driven, hypertrophic muscle. To date, work with ActRIIB-Fc and anti-MST antibodies suggests that building new muscle upon a dystrophic background may engender a measurable oxidative imbalance in the tissue, but not of enough magnitude to reduce force-generating capacity and fatigability [71–73].

Histopathological analysis of the tissues from the exercised study distinguished FS-EEE-mFc as capable of producing greater reductions in dystrophic pathology than the anti-MST antibody. In the limb muscles, the large hypertrophic response for FS-EEE-mFc at its high dose translated to reductions in necrosis and fibrosis of the quadriceps. In the diaphragm, the tissue in young *mdx* animals that displays the greatest degree of damage, FS-EEE-mFc reduced pro-inflammatory markers, suggesting that durable fibrosis reductions in dystrophic tissue may be achievable with a longer dosing regimen.

Follistatin does not act directly upon dystrophin and its therapeutic hypothesis rests on the assumption that hypertrophic and anti-inflammatory effects can combine to strengthen dystrophic muscle enough to slow progressive loss of limb and pulmonary muscle function. In *mdx* mice, greater hypertrophic responses are achievable than in larger animals, and this tempers translational conclusions that can be drawn from the *mdx* model. Whether hypertrophy alone is sufficient for therapeutic benefit in DMD will be informed by the outcomes from anti-myostatin agents currently in clinical development. Specifically, limb muscle imaging to assess pathological status will provide data to the question of healthiness of larger muscles in a dystrophic background.

Future studies can explore limb muscle pathology using a more damaging exercise regimen in *mdx* mice or other rodent models of limb injury and repair. Investigating the cardiopulmonary effects of follistatin treatment are warranted, employing either aged *mdx* mice or *mdx* mice on the DBA/2J strain background that reportedly displays more aggressive cardiac decline than the C57BL/10ScSnJ background [74].

## Conclusion

The hypertrophic and anti-fibrotic dual pharmacology of engineered follistatin provides an attractive therapeutic option for the treatment of dystrophic muscle disease.

## Abbreviations

AAV: Adeno-associated virus
CK: Creatine kinase
DMD: Duchenne muscular dystrophy
EDL: Extensor digitorum longum
hFc: Human fraction crystallizable (of IgG type 1)
HGP: Histidine-rich glycoprotein
IgG: Immunoglobulin G
IHC: Immunohistochemistry
IV: Intravenous route of administration
mFc: Mouse fraction crystallizable (of IgG type 1)
MST: Myostatin
PBS: Phosphate buffered saline
SC: Subcutaneous route of administration
TGF: Transforming growth factor
